# Plasmid Transfer of Plasminogen K1-5 Reduces Subcutaneous Hepatoma Growth by Affecting Inflammatory Factors

**DOI:** 10.1155/2014/656527

**Published:** 2014-05-08

**Authors:** Lea A. Koch, Volker Schmitz, Christian P. Strassburg, Esther Raskopf

**Affiliations:** ^1^Department of Inner Medicine I, University of Hospital Bonn, Sigmund-Freud-Straße 25, 53127 Bonn, Germany; ^2^Krankenhaus Marienwörth, Mühlenstrasse 39, 55543, Bad Kreuznach, Germany

## Abstract

There is evidence that plasminogen K1-5 (PlgK1-5) directly affects tumour cells and inflammation. Therefore, we analysed if PlgK1-5 has immediate effects on hepatoma cells and inflammatory factors *in vitro* and *in vivo*. *In vitro*, effects of plasmid encoding PlgK1-5 (pK1-5) on Hepa129, Hepa1-6, and HuH7 cell viability, apoptosis, and proliferation as well as VEGF and TNF-alpha expression and STAT3-phosphorylation were investigated. *In vivo*, tumour growth, proliferation, vessel density, and effects on vascular endothelial growth factor (VEGF) and tumour necrosis factor alpha (TNF-alpha) expression were examined following treatment with pK1-5. *In vivo*, pK1-5 halved cell viability; cell death was increased by up to 15% compared to the corresponding controls. Proliferation was not affected. VEGF, TNF-alpha, and STAT3-phosphorylation were affected following treatment with pK1-5. *In vivo*, ten days after treatment initiation, pK1-5 reduced subcutaneous tumour growth by 32% and mitosis by up to 77% compared to the controls. Vessel density was reduced by 50%. TNF-alpha levels in tumour and liver tissue were increased, whereas VEGF levels in tumours and livers were reduced after pK1-5 treatment. Taken together, plasmid gene transfer of PlgK1-5 inhibits hepatoma (cell) growth not only by reducing vessel density but also by inducing apoptosis, inhibiting proliferation, and triggering inflammation.

## 1. Introduction

Hepatocellular carcinomas (HCC) continue to pose a challenge in establishing effective therapies for a broad collective of patients. Novel therapeutic approaches such as sorafenib have been shown to inhibit HCC growth and prolong patient survival [1]. But in the light of possible escape mechanisms with monotarget treatments, a combination of antitumoral mechanisms may be preferable [2–4].

Angiostatin (PlgK1-4) and its derivate plasminogen K1-5 (PlgK1-5) are powerful angiostatic factors which inhibit endothelial cell functions* in vitro* and* in vivo* [5–8]. However, there has been mounting evidence that antitumor effects of angiostatin and its derivates go beyond effects on endothelial cells. Davidson et al. showed that kringle 5 alone induced not only apoptosis in endothelial cells but also in tumour cells as well [9]. This goes in line with a study by our workgroup also showing direct effects on hepatoma cells [10].

Additionally, there is evidence that antiendothelial and antitumoral effects of PlgK1-5 are complemented by a modulation of inflammatory pathways.

Albini et al. showed that angiostatin induces an upregulation of IL-12 in macrophages and concomitantly that the angiostatic properties are partly dependent on the IL-12 signal cascade [11]. IL-12 is a proinflammatory cytokine, supporting our theory that antitumor effects of angiostatin and its derivates are both angiostatic and proinflammatory. In line with this, Perri et al. found that kringle 5 (K5) supported the recruitment of neutrophils and NKT-cells [12], whereas Mauceri and coworkers demonstrated that tumour production of angiostatin was heightened after exposure to TNF-alpha [13].

It has been shown that heightened TNF-alpha levels correlate with elevated vascular endothelial growth factor (VEGF) levels [14, 15], whereas angiostatin has an antagonistic effect on VEGF [16, 17]. However, to our knowledge, this has not been shown for PlgK1-5.

Many studies used an adenoviral vector approach to investigate the antitumoral effects of PlgK1-5 [8, 10, 18, 19]. Yet adenovirus based therapies are prone to cause vehement allergic reactions in follow-up treatments [20].

Therefore, we analysed effects of plasmid vector mediated gene transfer of PlgK1-5 on hepatomas* in vitro* and* in vivo*. Since angiostatic effects are well documented, we placed our focus on other factors. We examined proapoptotic, antiproliferative, and proinflammatory pathways* in vitro* and* in vivo*. Additionally, we wanted to further distinguish the link between inflammation and angiogenesis.

## 2. Materials and Methods

### 2.1. Animals and Cell Lines

Eight-week-old male C3H mice were supplied by Charles River (Sulzfeld, Germany) and kept in the local central animal facility of the University Hospital Bonn. The mice were housed under standard conditions and had free access to water and food. Animal procedures were performed in accordance with approved protocols and followed recommendations for proper care and use of laboratory animals.

The hepatocyte derived cellular carcinoma cell line HuH7 and the mouse hepatoma cell line Hepa1-6 (both obtained from American Type Culture Collection (ATCC, Rockville, MD)) were cultivated in Dulbecco's modified Eagle medium (DMEM) supplemented with 10% heat-inactivated fetal bovine serum (FBS).

Hepa129 cells (Mouse Hepatoma 129, obtained from NCI-Frederick Cancer Research and Development Centre (DCT Tumour Repository)) were maintained in RPMI1640 supplemented with 10% FBS, 200 mM glutamine.

### 2.2. Synthesis and Amplification of pCMVTNT Vectors

Fragments encoding plasminogen K1-5 (PlgK1-5, amino acids 1-546) were generated and validated as described previously [8]. PlgK1-5 was then subcloned into the eukaryotic expression vector pCMVTNT (Promega, Mannheim, Germany) according to the manufacturer's protocol; correct insertion was determined through enzymatic restriction digestion and gel electrophoresis of pCMVTNT-K1-5 (pK1-5) on a 1% agarose gel. pCMVTNT without transgene (pMock) was used as a vector control.

pK1-5 and pMock were amplified using the E. coli K12 strain DH10B and plasmid vectors were isolated using the NucleoBond Xtra Maxi Plus EF system (Macherey-Nagel, Düren, Germany). DNA content and purity were determined by measuring the OD260/280 ratio.

### 2.3. Plasmid Transfection* In Vitro* and* In Vivo*

*In vitro*, 10^6^ cells (HuH7, Hepa1-6, Hepa129) were seeded on 10 cm plates and cultured in antibiotic-free medium. One day later, cells were transfected with either pK1-5 or pMock (*n* = 4 per group) using Lipofectamine 2000 (Invitrogen, Karlsruhe, Germany), according to the manufacturer's protocol. Cells treated with plain culture medium were used as negative controls.

For establishment of subcutaneous tumours, 10^6^ Hepa129 cells in 150 *μ*L plain RPMI were injected subcutaneously into the paramedian hind of male C3H mice [8]. Reaching a mean tumour diameter of 150 cm^3^, treatment was started. In a two-day interval, mice were treated with intraperitoneal injections with NaCl (150 *μ*L, NaCl/mouse, *n* = 5), DOTAP (Carl Roth, Karlsruhe, Germany) alone (100 *μ*L NaCl with 50 *μ*L DOTAP per animal, *n* = 6), pMock (50 *μ*g pMock in 100 *μ*L NaCl and 50 *μ*L DOTAP, *n* = 6), or pK1-5 (50 *μ*g pK1-5 in 100 *μ*L NaCl with 50 *μ*L DOTAP, *n* = 5).

### 2.4. Analysis of K1-5 Protein Expression* In Vitro* and* In Vivo*

To determine K1-5 expression* in vitro,* cells were transfected as described above. Two days later the cells were harvested in 100 *μ*L PBS with protease inhibitors (Complete, Roche Diagnostics, Mannheim, Germany). For analysis of K1-5 expression* in vivo*, liver and tumour tissues (50 mm^3^) were harvested after termination of the* in vivo* experiments. Tumour and liver tissues were homogenised in 500 *μ*L PBS and protease inhibitors (Complete) using the Precellys system (Peqlab, Heidelberg, Germany). Cells and tissue homogenisates were then lysed by repeated freeze-thaw cycles. Protein concentrations of tissue and cells were determined using the DC protein assay (Biorad, Munich, Germany) according to the manufacturer's protocol.

50 *μ*g protein was used for SDS-polyacrylamide gel electrophoresis. Proteins were transferred on PVDF membrane (Biorad). Detection of K1-5 was done using an anti-angiostatin antibody (A1101, Sigma-Aldrich, Hamburg, Germany) and a corresponding secondary antibody (rabbit anti-goat, Santa Cruz, Heidelberg, Germany). GAPDH was used as a control (sc-25778, Santa Cruz). Chemiluminescent signal was developed using AceGlow (Peqlab) and the Chemismart system with ChemiCapt software (Peqlab).

### 2.5. Determination of Tumour Growth* In Vivo* and Sample Collection

Tumour growth was determined every second day by measuring maximum length and width using a caliper. Tumour volumes were calculated as *V* = length × (width)^2^  × 0.52.

Ten days after treatment initiation, mice were sacrificed and tumours and livers were explanted. Blood was collected. Tumour, liver, and blood samples were harvested and conserved at −80°C after freezing in liquid nitrogen. Tumour samples were embedded in Tissue Tek (Sakura Finetek, Heppenheim, Germany) and cryopreserved for immunohistochemistry.

### 2.6. Determination of Cell Viability

One day prior to transfection, 10^4^ cells were seeded onto 96-well-plates and then cultivated in antibiotic-free medium. 100 *μ*L 3-(4,5-dimethylthiazol-2-yl)-2,5-diphenyltetrazolium bromide (MTT) was added to the cells 48 hours after transfection. Cells were then incubated for 45 minutes. Supernatants were removed and MTT was solubilized with 100 *μ*L dimethyl sulfoxide (DMSO). Optical density was measured colorimetrically at 560 nm (GloMax Multi ELISA reader, Promega, Mannheim, Germany).

To test for effects on tumour cell proliferation, cells were labelled using 5-bromo-2′-deoxyuridine (BrdU) in accordance with the manufacturer's protocol (Cell Proliferation ELISA, Roche Diagnostics, Mannheim, Germany) 24 hours after transfection. Another 24 hours later, cells were fixed and BrdU incorporation was determined using a specific antibody. Optical density was determined at 450 nm (GloMax Multi ELISA reader).

Apoptosis was determined by quantifying cytoplasmic histone-associated DNA fragments. The Cell Death Detection kit (Roche Diagnostics) was applied following the manufacturer's protocol. 48 hours after transfection, cells were lysed and histone-associated DNA was determined using specific antibodies and ELISA technique. Optical density was determined at 405 nm (GloMax Multi ELISA reader).

### 2.7. Cell-Based Phospho-STAT3 Immunoassay

For analysis of (phosphorylated) STAT3, 10^4^ cells were seeded on a black 96-well tissue culture plate with clear bottom. The cells were transfected as described above. Two days after transfection, STAT3 phosphorylation was quantified with a cell-based STAT3 Immunoassay (R&D Systems, Wiesbaden, Mannheim, Germany) as described in the manufacturer's protocol. STAT3 and phosphorylated STAT3 (pSTAT3) were detected with two different specific antibodies and corresponding fluorescent secondary antibodies.

Fluorescence was measured with the GloMax Multi ELISA reader (Promega). For STAT3, fluorescence was measured at 360 nm (excitation) and 450 nm (extinction) and at 540 nm (excitation) and 600 nm (extinction) for pSTAT3.

### 2.8. Analysis of VEGF and TNF-Alpha Levels* In Vitro* and* In Vivo*

Cells were transfected and harvested as described in the passage about the detection of K1-5 protein. Tumour and liver tissues were homogenised as described above. Cells and tissue homogenisates were then lysed by repeated freeze-thaw cycles. Protein concentrations of tissue and cells were determined using the DC protein assay (Biorad, Munich, Germany) according to the manufacturer's protocol.

VEGF in HuH7 was quantified with the human VEGF DuoSet ELISA kit (R&D Systems, Wiesbaden, Germany). VEGF in Hepa1-6, Hepa129, and liver and tumour tissue samples was analysed with the murine VEGF DuoSet ELISA kit (R&D Systems) as instructed. VEGF levels in cell supernatants were correlated with the cell viability as established in the MTT assay.

TNF-alpha in HuH7 was analysed with the human TNF-alpha DuoSet ELISA kit (R&D Systems). TNF-alpha in Hepa1-6, Hepa129, and tumour and liver tissues was quantified with the murine TNF-alpha DuoSet ELISA kit (R&D Systems).

### 2.9. Histological Sections and Staining

Cryopreserved tumour samples were standard HE stained.

Immunostaining against CD31 was done with monoclonal rat anti-mouse CD31 (Cedarlane, Ontario, Canada) and Ki67 staining with rat anti-mouse Ki67 (DakoCytomation, Hamburg, Germany) according to a previous publication [10].

For quantification of CD31 positive blood vessels and Ki67 positive mitotic cells, microscopic fields of vision (= high power fields, hpf) were evaluated.

Sections were stained against VEGF with goat anti-mouse VEGF (R&D Systems). For staining against TNF-alpha, goat anti-mouse TNF-alpha (Abcam, Cambridge, UK) was used.

Sections were incubated with corresponding secondary antibodies (DakoCytomation). Detection was done using streptavidin and AEC substrate (DakoCytomation). Sections were counterstained with hematoxyline.

### 2.10. Statistical Analysis

Data are presented as means with SEM. Statistically significant differences between experimental groups were shown by using a nonparametric, two-tailed test (Mann-Whitney test) for unpaired samples. Values of *P* < 0.05 were considered to be significant.

## 3. Results

### 3.1. PlgK1-5 Is Expressed* In Vitro* and* In Vivo*

First, we analysed PlgK1-5 protein expression following transfection with pK1-5* in vitro* and* in vivo*.

**Figure 1:**
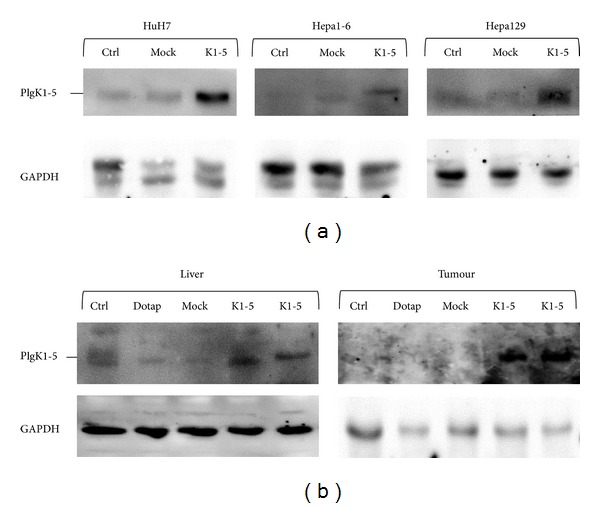
Protein expression of PlgK1-5* in vitro* and* in vivo*. Cells were transfected with pMock or pK1-5 and incubated for 48 hours. Cells were harvested, and protein was isolated. Tumour and liver tissue fragments from mice treated with NaCl, DOTAP alone, pMock, or pK1-5 were homogenised using the Precellys system and protein was isolated. Whole protein content was determined and 50 *μ*g protein was subjected to SDS-PAGE. Following semidry blotting, PlgK1-5 was detected using an angiostatin-specific antibody. GAPDH was used as a control. Ctrl: NaCl or plain medium control; Dotap: DOTAP control; Mock: pMock; K1-5: pK1-5.

Using western blot technique, PlgK1-5 was detected in all three analysed hepatoma cell lines transfected with pK1-5, whereas in the pMock and medium control, PlgK1-5 was barely detected. This was also the case for subcutaneous tumours and corresponding livers. PlgK1-5 was stronger expressed in animals receiving pK1-5. In the control groups, bands were only slightly visible (Figure 1).

### 3.2. pK1-5 Reduces Cell Viability by Increasing Cellular Death but Has Little Impact on Cell Proliferation

After showing PlgK1-5 expression after pK1-5 treatment, we examined whether pK1-5 affects overall cell viability, cell proliferation, and cell death in hepatoma cell lines.

**Figure 2:**
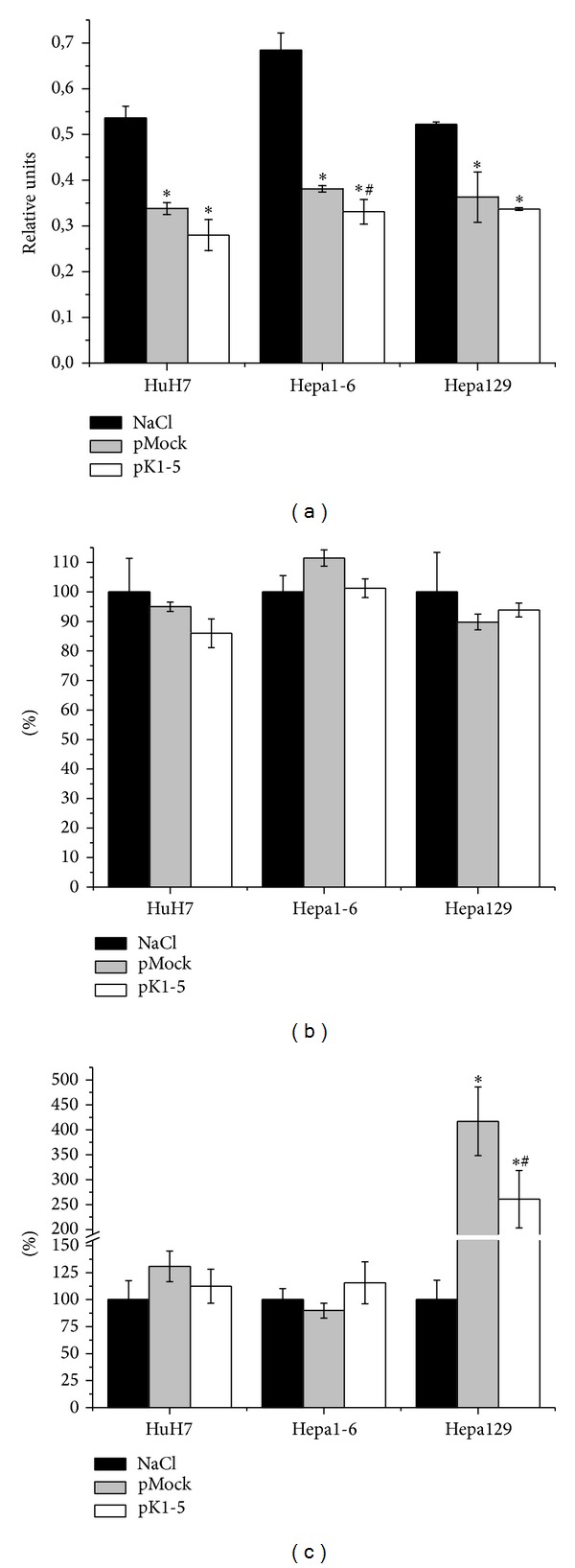
K1-5 and cell viability. 10^4^ hepatoma cells were seeded on 96-well plates, transfected with Lipofectamine and pCMVTNT-vectors or treated with plain medium and incubated for twenty four hours; *n* = 4, *P < 0.05 (plain medium/pK1-5), ^#^ *P* < 0.05 (pMock/pK1-5). Optical density of medium treated cells was set as 100%. Data are shown as mean percentage ± SEM. (a) Supernatants were removed, and MTT was added and incubated. Supernatants were removed, MTT was solubilized with DMSO, and OD was measured at 560 nm. (b) Cells were labeled with BrdU, incubated for 24 hours, and fixed with a specific antibody and optical density was measured at 560 nm. (c) Cytoplasmatic histone-associated DNA fragments were quantified with the Cell Death Detection kit (Roche diagnostics) and optical density measured at 405 nm.

In an MTT assay, pK1-5 reduced cell viability in all three examined cell lines. HuH7 treated with pK1-5 were 10.91% (*n* = 4, *P* = 0.0286) less viable than pMock treated cells. Compared to the medium control, viability was reduced by 47.86% (*n* = 4, *P* = 0.0286). In pK1-5 treated Hepa1-6, viability was reduced by 7.36% (*n* = 4, n.s.) compared to pMock and by 51.58% compared to the medium control (*n* = 4, *P* = 0.0286). When treated with pK1-5, Hepa129 showed 4.96% less viability than pMock treated cells (*n* = 4, n.s.). Viability was decreased by 35.37% when compared to the medium control (*n* = 4, *P* = 0.0286, Figure 2(a)).

To ascertain whether reduced cell viability was due to decreased cell proliferation, we examined effects of pK1-5 on BrdU incorporation in HuH7, Hepa1-6, and Hepa129. When treated with pK1-5, proliferation of HuH7 was reduced by 9.04% compared to pMock treated cells (*n* = 4, n.s.) and by 14.03% compared to the medium control (*n* = 4, *P* = 0.0286). In Hepa1-6, pK1-5 treated cells showed 10.21% less proliferation than pMock treated cells (*n* = 4, n.s.) but showed no effect compared to the medium control (*n* = 4, *P* = 0.0286, Figure 2(b)).

To analyse if diminished cell viability is due to increased apoptosis, we applied a cell death detection assay. HuH7 treated with pK1-5 showed 18.33% less apoptotic cells than HuH7 treated with pMock (*n* = 4, n.s.) but 12.5% higher rates of apoptosis compared to the medium control (*n* = 4, n.s.). When treated with pK1-5, apoptosis in Hepa1-6 was increased by 25.75% compared to pMock treated cells (*n* = 4, n.s.) and by 15.62% compared to the medium control (*n* = 4, n.s.). In Hepa129, apoptosis was reduced by 37.46% in pK1-5 treated cells compared to the pMock group (*n* = 4, n.s.) but increased 2.6-fold compared to the medium control (*n* = 4, *P* = 0.0286, Figure 2(c)).

### 3.3. pK1-5 Increases VEGF Expression and Secretion in Hepatoma Cells* In Vitro*

To test whether pK1-5 affects VEGF expression, a VEGF ELISA was performed.

**Figure 3:**
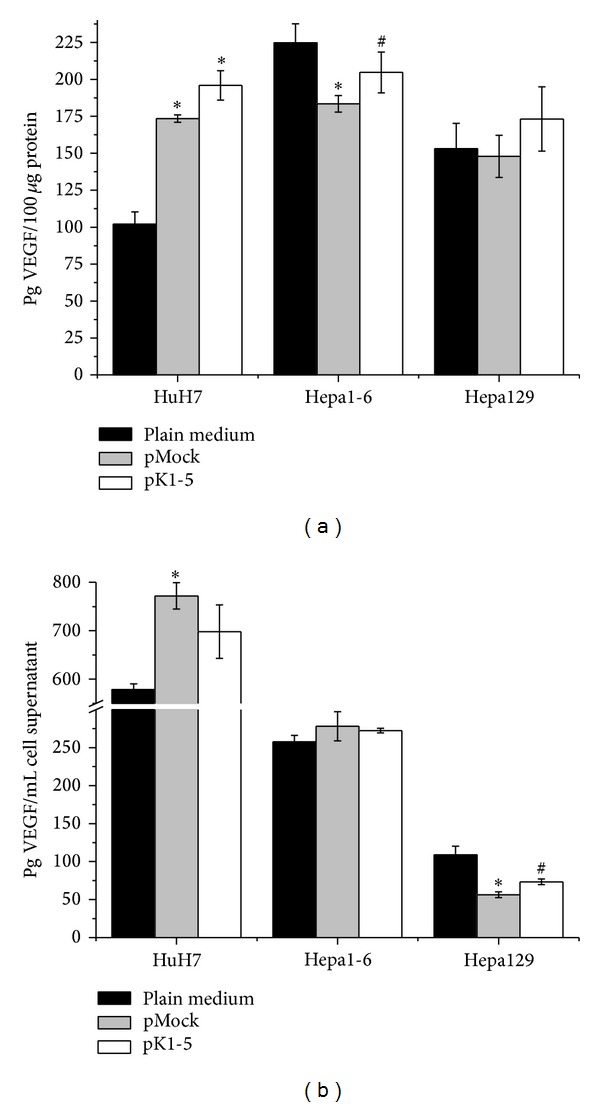
VEGF expression after pK1-5 treatment* in vitro*. Cells were transfected and incubated for 48 hours. Cells and supernatants were harvested, and cells were solubilized in PBS/complete and lysed with repeated freeze-thaw cycles. Protein levels were analysed and VEGF levels were quantified with human (HuH7) or murine (Hepa1-6, Hepa129) VEGF ELISAs. VEGF levels in supernatants were correlated to OD acquired by MTT assays. (a) VEGF levels in cell lysates. Data are shown as mean pg VEGF/100 *μ*g protein ± SEM. (b) VEGF levels in supernatants, MTT correlated. Data are shown as mean pg VEGF/mL cell supernatant ± SEM. *n* = 4, *P < 0.05 (compared to plain medium), ^#^ *P* < 0.05 (compared to pMock).

In detail, VEGF expression in pK1-5 treated HuH7 was raised by 12.97% compared to pMock treated HuH7 (*n* = 4, n.s.) and 1.9-fold compared to the medium control (*n* = 4, *P* = 0.0286). In Hepa1-6, VEGF levels in the pK1-5 group were elevated compared to pMock treated cells by 11.59% (*n* = 4, n.s.) but slightly lowered by 8.93% compared to medium treated Hepa1-6 (*n* = 4, n.s). In Hepa129, treatment with pK1-5 increased VEGF expression by 17.12% with regards to the pMock group (*n* = 4, n.s.) and by 13.13% compared to the medium control (*n* = 4, n.s., Figure 3(a)).

Correlated with cell viability, pK1-5 reduced VEGF secretion in HuH7 by 9.55% compared to pMock treated cells (*n* = 4, n.s.) but increased secretion by 20.65% compared to medium treated HuH7 (*n* = 4, n.s.). In Hepa1-6, pK1-5 appeared to have no significant effect on VEGF secretion. VEGF levels in supernatant of pK1-5 treated cells were increased by 5.73% (*n* = 4, n.s.) compared to the medium control but decreased by 2.09% compared with pMock (*n* = 4, n.s.).

Hepa129 treated with pK1-5 secreted 30.42% more VEGF than Hepa129 treated with pMock (*n* = 4, n.s.) but expressed 32.6% less than the medium control (*n* = 4, *P* = 0.0286, Figure 3(b)).

### 3.4. TNF-Alpha Is Increased in pK1-5-Treated Hepatoma Cells

Since VEGF was affected by pK1-5, we wanted to know if this was due to TNF-alpha.

**Figure 4:**
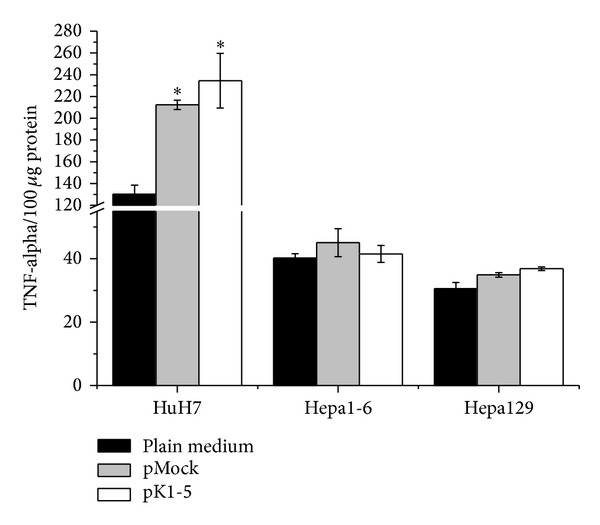
TNF-alpha expression after pK1-5 treatment* in vitro*. 10^6^ cells were seeded on 10 cm plates and transfected with Lipofectamine and pCMVTNT-vectors or plain medium and incubated for 48 hours. Cells were harvested, solubilised in PBS/complete, and treated with repeated freeze-thaw cycles and protein levels were analysed. TNF-alpha was quantified with human (HuH7) or murine (Hepa1-6, Hepa129) TNF-alpha ELISAs. Data are shown as mean pg TNF-alpha/100 *μ*g protein ± SEM. *n* = 4, *P < 0.05 (compared to plain medium).

In all three investigated cell lines, TNF-alpha was elevated following treatment with pK1-5 (Figure 4). In HuH7, cells treated with pK1-5 (*n* = 4) expressed 44.54% more TNF-alpha than the medium control (*n* = 4, *P* = 0.0286) and 9.46% more than cells treated with pMock (*n* = 4, n.s.).

Hepa1-6 treated with pK1-5 (*n* = 4) produced 3.11% more TNF-alpha than the medium control (*n* = 4, n.s.), but 8.58% less than cells treated with pMock (*n* = 4, n.s.).

In pK1-5 treated Hepa129 (*n* = 4), TNF-alpha was increased by 17.31% when compared to medium treated cells (*n* = 4, n.s.). When compared to pMock, pK1-5 treated Hepa129 expressed 5.35% more TNF-alpha (*n* = 4, n.s.).

### 3.5. STAT3 and pSTAT3 Expression Is Modulated by pK1-5

Signal transducer and activator of transcription 3 (STAT3) is an acute-phase response factor that is activated through phosphorylation. To detect possible effects of pK1-5 on cell growth and further elucidate pK1-5 effects on inflammation, we analysed STAT3 phosphorylation. We found that both total STAT3 expression and phosphorylation were altered by treatment with pK1-5 in all tested cell lines.

**Figure 5:**
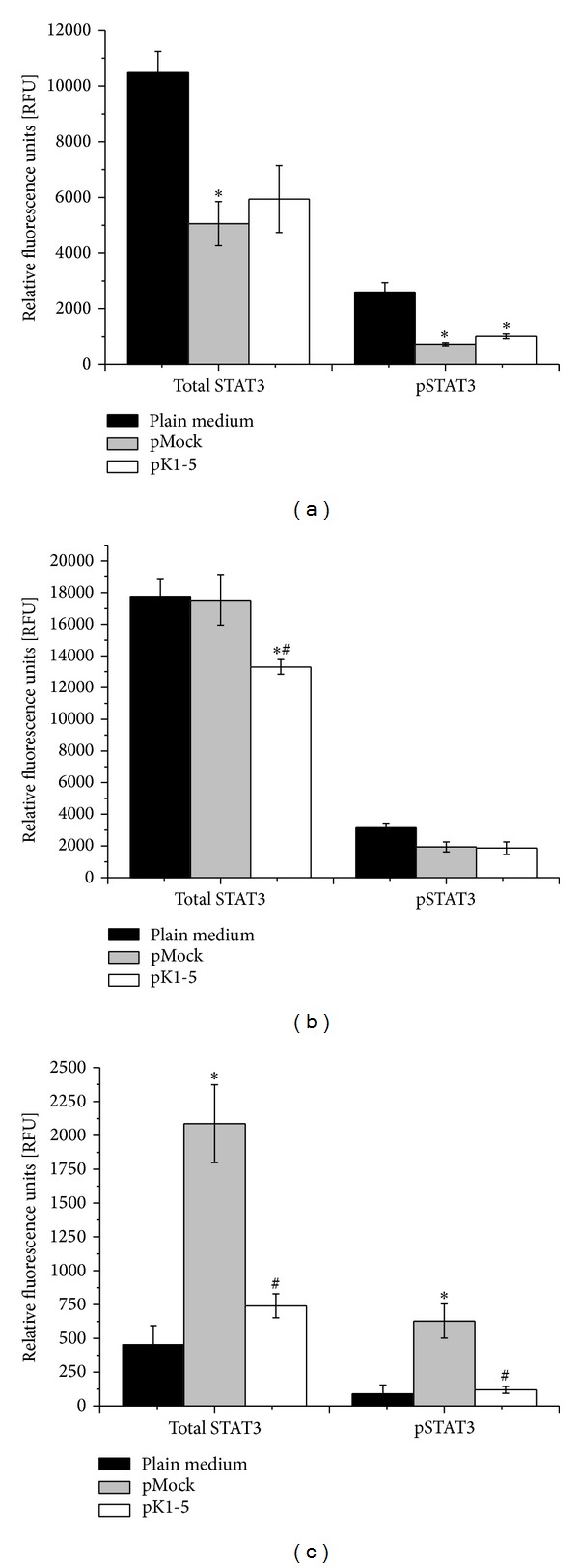
STAT3 phosphorylation. 10^4^ HuH7 (a), Hepa1-6 (b), or Hepa129 (c) were seeded on a sterile black 96-well tissue plate with clear bottom, transfected with Lipofectamine and pCMVTNT vectors, or treated with plain medium and incubated for 48 hours. STAT3 phosphorylation was quantified according to manufacturer's protocol (STAT3 immunoassay, R&D systems). Fluorescence was measured with the GloMax Multi (Promega) ELISA reader. For STAT3, fluorescence was measured at 360 nm (excitation) and 450 nm (extinction) and at 540 nm (excitation) and 600 nm (extinction) for pSTAT3. Data are presented as relative fluorescence units ± SEM. *n* = 4, *P < 0.05 (compared to plain medium), ^#^ *P* < 0.05 (compared to pMock).

In HuH7 cells treated with pK1-5 expressed 76.45% less total STAT3. In comparison with pMock treated cells, pK1-5 increased total STAT3 by 14.95%. Levels of pSTAT3 were reduced by 61.02% compared to the medium control. Compared with pMock, pK1-5 increased pSTAT3 by 28.53% (Figure 5(a)).

When treated with pK1-5, Hepa1-6 produced 33.51% less total STAT3. Compared to pMock, pK1-5 reduced total STAT3 levels by 31.73%. pK1-5 treated Hepa1-6 showed 68.73% less pSTAT3 in comparison to the medium control. Compared to pMock, pK1-5 reduced pSTAT3 levels by 4.33% (Figure 5(b)).

Hepa129 treated with pK1-5 showed increased total STAT3 (by 38.77%) and phosphorylation of STAT3 (by 24.81%) compared to the medium control. When compared with the pMock control, total STAT3 levels were reduced by 64.51% and pSTAT3 was decreased by 80.96% lower when treated with pK1-5 (Figure 5(c)).

### 3.6. Subcutaneous Tumour Growth Is Strongly Reduced in pK1-5 Treated Mice

After showing that pK1-5 affects tumour cells* in vitro*, we wanted to investigate whether these effects could be transferred in a subcutaneous tumour model.

**Figure 6:**
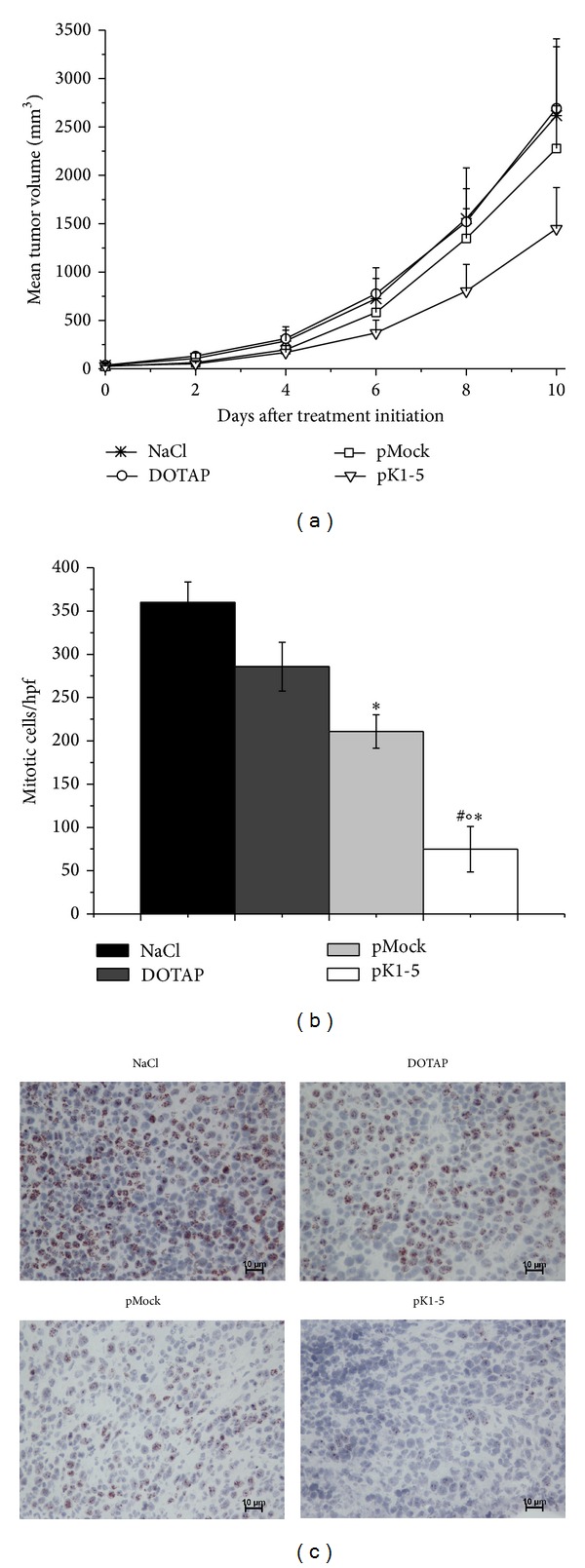
Antiproliferative and proapoptotic effects of pK1-5 in murine subcutaneous tumours: (a) tumour development after treatment with NaCl (*n* = 5), DOTAP (*n* = 6), pMock (*n* = 6), or pK1-5 (*n* = 5). 10^6^ Hepa129 were injected subcutaneously into the paramedian hind of male C3H mice. Treatment was started when tumours reached a mean volume of 150 mm^3^ with subcutaneous injections every second day. Tumour size was measured every second day using a caliper and volumes were calculated as *V* = length × (width)^2^  × 0,52. Data are presented as the mean tumour volume in mm^3^ ± SEM. (b) Mitosis in Ki67 immunostained sections. Tumour sections were immunostained against Ki67 and counterstained with hematoxyline. Mitotic cells were counted per hpf (*n* = 16). Data are shown as mitotic cells ± SEM. *P < 0.05 (compared to NaCl), °*P* < 0.05 (compared to DOTAP), ^#^ *P* < 0.05 (compared to pMock). (c) Exemplary photomicrographs of animals treated with NaCl, DOTAP, pMock, pK1-5. Mitotic cells show a brown nuclear staining.

Ten days after treatment initiation, tumour growth was reduced by 22.36% in pK1-5 treated mice when compared to pMock treated animals. Compared to the DOTAP control, tumour growth of pK1-5 treated animals was reduced by 34.44% and by 32.44% in comparison to NaCl treated mice (Figure 6(a)).

In order to differentiate whether inhibitory effects of pK1-5 on tumour growth were due to increased cell death and/or reduced proliferation, we analysed tumour sections for necrosis and mitosis.

pK1-5 (*n* = 4) reduced necrotic areas by 37% ± 23.64% compared to pMock (*n* = 4, n.s.) and by 22% ± 8.16% compared to DOTAP controls (*n* = 4, n.s.). Necrotic areas of sections of pK1-5 treated animals were reduced by 82% ± 24.5% compared to NaCl (*n* = 4, *P* = 0.0286), as determined by HE staining (data not shown).

Ki67 staining revealed that pK1-5 reduced mitosis by 68.4% compared to pMock treated animals and by 73.49% in the DOTAP control. Mitotic cells in pK1-5 treated mice were decreased by 77.37% compared with NaCl controls (Figures 6(b) and 6(c)).

### 3.7. pK1-5 Affects VEGF Expression* In Vivo*

VEGF levels* in vitro* had been elevated following treatment with pK1-5. In order to examine pK1-5 effects on VEGF levels* in vivo*, we performed a VEGF ELISA with tumour and liver tissues.

**Figure 7:**
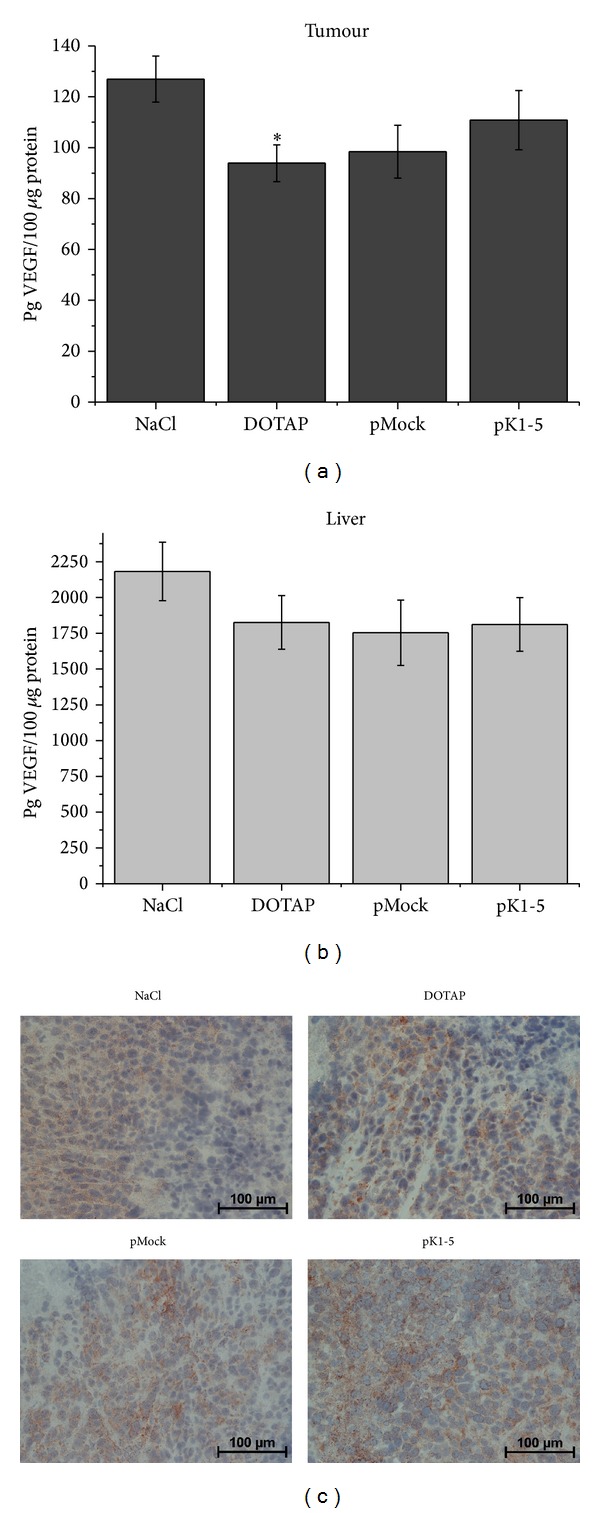
*In vivo* effects of pK1-5 on VEGF expression. Mice with subcutaneous tumours were treated with NaCl (*n* = 5), DOTAP (*n* = 6), pMock (*n* = 6), or pK1-5 (*n* = 5) and sacrificed after ten days. Tumours and livers were explanted and homogenized, serum was collected, and protein concentrations were evaluated. VEGF levels were quantified with a murine VEGF ELISA. Data are presented as mean pg VEGF ± SEM. *P < 0.05 (compared to NaCl). (a) Tumour samples, (b) liver samples, and (c) tumour sections were stained against VEGF and counterstained with hematoxyline. Exemplary photomicrographs of animals treated with NaCl, DOTAP, pMock, and pK1-5. VEGF is shown by brown staining.

VEGF levels in tumour samples of pK1-5 treated animals were reduced by 12.67% in comparison to the NaCl control but heightened when compared to the DOTAP control (+18.08%) and pMock control (+12.63%, Figure 7(a)). This was confirmed by immunohistochemistry (Figure 7(c)). VEGF levels in liver samples were not altered in the pK1-5 group compared to pMock and DOTAP treated mice but reduced by 17% compared to the NaCl control (Figure 7(b)).

### 3.8. TNF-Alpha Expression* In Vivo* Is Altered by pK1-5

**Figure 8:**
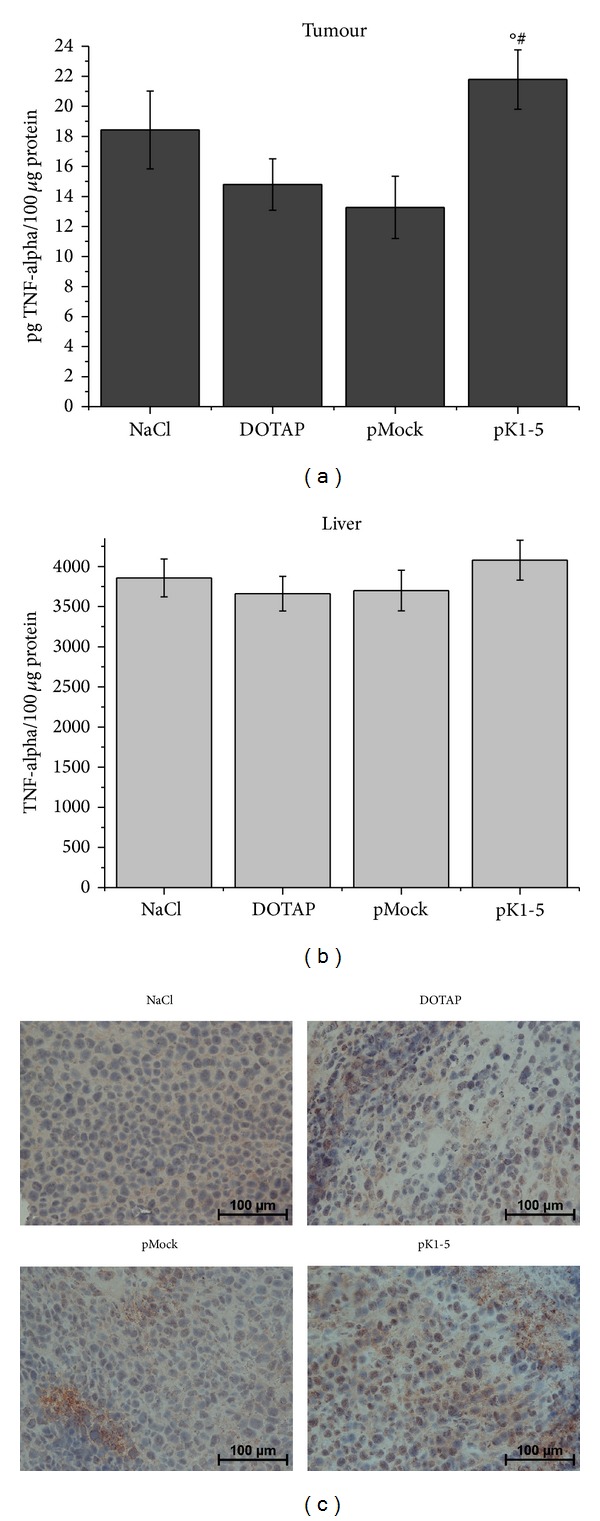
*In vivo* effects of pK1-5 on TNF-alpha expression. Mice with subcutaneous tumours were treated with NaCl (*n* = 5), DOTAP (*n* = 6), pMock (*n* = 6), or pK1-5 (*n* = 5) and sacrificed after ten days. Tumours and livers were explanted and homogenized and protein concentrations were evaluated. TNF-alpha levels were quantified with a murine TNF-alpha ELISA. Data are presented as mean pg TNF-alpha/100 µg protein. °*P* < 0.05 (compared to DOTAP), ^#^ *P* < 0.05 (compared to pMock). (a) Tumour samples, (b) liver samples, and (c) tumour sections were stained against TNF-alpha and counterstained with hematoxyline. Exemplary photomicrographs of animals treated with NaCl, DOTAP, pMock, and pK1-5. TNF-alpha is shown by brown staining.

Following treatment with pK1-5, intratumoural TNF-alpha levels were increased by 18.2% in comparison to the NaCl control. In the DOTAP and the pMock control, TNF-alpha concentrations were decreased compared to the NaCl control by 19.69% and by 27.98%, respectively. TNF-alpha levels in the pK1-5 group showed a significant increase compared to the DOTAP (+47.18%) and the pMock group (+64.13%, Figure 8(a)).

Mice treated with pK1-5 showed altered TNF-alpha expression in livers, as well. When compared with pMock treated mice, pK1-5 increased TNF-alpha levels by 10.22%. Compared to DOTAP, pK1-5 treated mice showed 11.41% higher TNF-alpha. Comparing NaCl treated mice with pK1-5 treated animals, pK1-5 increased TNF-alpha expression in livers by 5.75% (Figure 8(b)).

Elevated intratumoural TNF-alpha levels were supported by TNF-alpha staining in tumour sections. Intensity of staining indicating presence of TNF-alpha was noticeably increased in sections of pK1-5 treated mice compared to the controls (Figure 8(c)).

### 3.9. pK1-5 Strongly Reduces Vessel Density in Subcutaneous Hepatomas

Angiostatin and its derivate PlgK1-5 have already been shown in various studies to be potent angiostatic factors [8, 21]. The focus of this study was therefore not on antiangiogenesis. Nevertheless, we performed CD31 staining of tumour sections to demonstrate the angiostatic potency of pK1-5.

**Figure 9:**
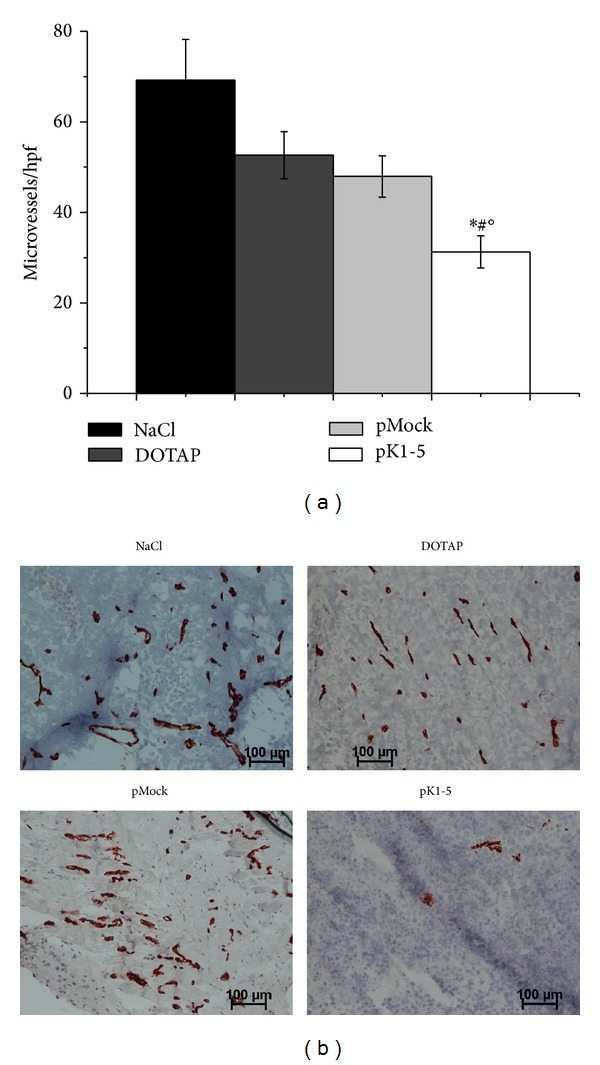
Microvessel density in subcutaneous tumours. Tumours of killed mice were explanted, shock frozen, stained against CD31, and counterstained against hematoxyline. Vessels show a red-brownish colour. (a) Mean tumour vessels ± SEM/hpf, *n* = 16, *P < 0.05 (compared to NaCl), °*P* < 0.05 (compared to DOTAP), ^#^ *P* < 0.05 (compared to pMock). Microvessels per hpf were microscopically counted. (b) Exemplary photomicrographs of tumour sections from animals treated with NaCl, DOTAP, pMock, and pK1-5.

CD31 staining showed that intratumoural microvessels were reduced by 54.83% in pK1-5 treated mice when compared to the NaCl control. In comparison with DOTAP treated mice pK1-5 treated animals had 40.62% less intratumoural vessels and showed 34.81% less vessels than pMock treated mice (Figure 9).

## 4. Discussion

To this day, hepatocellular carcinomas (HCC) belong to the most frequent malignant neoplastic diseases worldwide [22, 23]. Yet despite of new developments, therapeutic options are still rare and prognosis of patients in advanced stages remains poor [24].

In our opinion it is achievable that molecular treatment strategies should not only aim at one specific molecule—which could lead to escape mechanisms [2]—but rather inhibit multiple targets. This is mainly accomplished by combining therapies, but a single adjuvant with, for example, angiostatic and inflammatory properties would be preferable.

The angiostatic potency of angiostatin and its derivate PlgK1-5 have been shown in various studies [25–27]. But beyond that, recent data showed that angiostatin and PlgK1-5 not only affected endothelial cells but tumour cells as well [9, 10, 28].

Adenoviral gene therapies are viewed critically because of restrictions on follow-up treatments due to their high allergic potential [20, 29], and hydrodynamic injections practised in some mouse models are potentially fatal in humans. Previous studies already demonstrated that a plasmid based gene transfer of angiostatin and its derivates is effective [30–32]. For this reason, we applied a eukaryotic plasmid encoding PlgK1-5.

In this study, we demonstrated that pK1-5 strongly reduced cell viability in three different human and murine hepatoma cell lines. On further examination, we showed that reduced viability was caused by increased cell death through apoptosis. This confirms the antitumoral potential of PlgK1-5, as was already demonstrated in previous studies by our workgroup: adenoviral mediated gene transfer of PlgK1-5 reduced the expression of ICAM on murine (Hepa129) and human (HuH7) hepatoma cells, which led to heightened apoptosis [10]. This is also in accordance with prior findings by Davidson et al. Kringle 5 alone increased apoptosis in D54 human glioma tumour cells [9]. Induction of apoptosis could be due to the inhibition of mitochondrial proteins, as was shown by a study by Lee et al. Angiostatin downregulated BCL-2 protein, resulting in the activation of apoptotic pathways in human A2058 melanoma and BxPC-3 pancreatic tumour cell lines [28]. This supports our hypothesis that pK1-5 activates apoptotic pathways in hepatoma cells.

A previous study showed that antiendothelial and antitumoral effects of plasminogen kringle 5 are supported by proinflammatory mediators such as VCAM-1 and E-selectin [12]. Therefore, we analysed if TNF-alpha and VEGF, which link inflammation and angiogenesis, were also affected by pK1-5 treatment.

We found that TNF-alpha was heightened in all three examined hepatoma cell lines indicating a proinflammatory potential of pK1-5. This goes in line with a study by Perri et al., showing that retroviral vector based transduction of kringle 5 activated proinflammatory pathways resulting in decreased tumour growth in murine mammary adenocarcinoma (DA/3) cells [12]. We found that in two out of the three examined hepatoma cell lines, STAT3 expression and STAT3 phosphorylation as markers for immune responses were strongly reduced following pK1-5 treatment. The downregulation of STAT3 expression corresponds to the reduction of cell viability as shown in the MTT assay. Both TNF-alpha and STAT3 are key actors that are regulated through various mechanisms [33–35]; thus the exact mechanism of pK1-5 effects on either remain to be elucidated. Therefore, it is also possible that STAT3 expression and phosphorylation are activated through mediators other than pK1-5. Signal transduction leading to inflammatory responses should be further investigated; this study gives first indications that pK1-5 activates inflammation via an upregulation of TNF-alpha.

In previous studies, raised TNF-alpha levels correlated with heightened VEGF levels [14, 15]. VEGF is a key mediator in tumour angiogenesis, and for this reason, we examined pK1-5 effects on VEGF expression* in vitro*, evaluating both inflammatory and angiostatic capacities of pK1-5 mediated through VEGF. We found that, in cell lysates, VEGF levels were considerably heightened. In cell supernatants correlated to viability, VEGF levels were raised in two out of three examined hepatoma cell lines.

Our findings of increased VEGF levels despite of the angiostatic potency of PlgK1-5 can be explained by the induction of TNF-alpha. Here, the angiostatic and proinflammatory potency of PlgK1-5 counteracts the proangiogenic effects of VEGF.

Additionally, previous studies showed that VEGF itself plays a role in inflammation [36, 37]. We postulate that through this pathway, pK1-5 activates inflammation in hepatoma cells through an induction of TNF-alpha and increases this proinflammatory impact via a TNF-alpha triggered upregulation of VEGF.

After showing the proapoptotic and proinflammatory effects of pK1-5* in vitro,* we wanted to validate these results in a subcutaneous mouse model.

In one of our own studies, we already demonstrated that adenoviral mediated gene transfer of PlgK1-5 led to drastically reduced tumour growth and blood vessel density [8]. Corresponding to this, we showed in the study presented here that also plasmid based gene transfer of PlgK1-5 decreased subcutaneous tumour growth. The inhibition of experimental tumour growth of, for example, murine adenocarcinoma or human glioma cells after the treatment with angiostatin or its derivates, for example, via retroviral or adenoviral gene transfer had already been described in previous studies, going in line with our findings [7, 12, 19].

Tumour cell proliferation was reduced in our setting, confirming previous findings that PlgK1-5 exerts antiproliferative capacities in hepatoma and other tumour entities [5, 9, 10]. In contrast to that, necrosis found in HE stained sections of animals treated with PlgK1-5 was decreased compared to the NaCl control. This could be caused by markedly lessened tumour growth and mitosis, resulting in a decrease of apoptotic pressure on the tumour cells.

In addition to reduced tumour growth, expression of proangiogenic/proinflammatory factors like VEGF and TNF-alpha was affected by pK1-5. Corresponding to the* in vitro* experiments, TNF-alpha levels in tumour and liver tissue were heightened.

While we found a correlation between heightened TNF-alpha levels and increased VEGF expression* in vitro*, VEGF was decreased in tumour and liver tissue. This discrepancy of VEGF levels* in vitro* and* in vivo* may be caused by the considerable reduction of tumour growth and mitotic active cells, resulting in a lower number of VEGF secreting cells. Furthermore, PlgK1-5 has angiostatic potencies that may play a role in reducing* in vivo* VEGF levels. Additionally, angiostatin is an antagonist of VEGF [38, 39]. But so far, the exact link between TNF-alpha and VEGF regarding PlgK1-5 remains to be found.

In this context, two different approaches to further elucidate pK1-5 and its role in inflammation and reduced tumour progression appear promising. One step could be to evaluate the recruitment of inflammatory cells such as T-cell lymphocytes, NK-cells, and macrophages. This could be extended with an analysis of cell adhesion molecules modulating leukocyte and neutrophil recruitment such as PECAM-1 and ICAM or VCAM. First hints that PlgK1-5 affects these proinflammatory factors came from a study by our workgroup [10]. Another access to a further insight into inflammatory responses could be the expression of cytokines such as IL-12. The role of IL-12 in tumour suppression has already been shown by Albini and coworkers, who found that angiostatin-induced upregulation of IL-12 is an important mechanism behind angiostasis [11]. In our study, we placed the focus on inflammation and apoptosis rather than antiangiogenesis. Nevertheless, we evaluated microvessel density to demonstrate that plasmid based gene transfer of PlgK1-5 exhibits angiostatic effects comparable to an adenoviral based gene transfer [5, 7]. As expected, we found a strong reduction of microvessel density in animals treated with pK1-5. This demonstrates that antiangiogenic capacities of PlgK1-5 are not limited to adenoviral gene transfer.

In summary, we found that plasmid vector based gene transfer of PlgK1-5 had marked proapoptotic and proinflammatory effects on hepatoma cells* in vitro* and* in vivo* in addition to the predescribed effects on endothelial cells. This supports our theory that pK1-5 not only has direct apoptotic and antiproliferative effects on hepatoma cells but also leads to inflammation in addition to the known angiostasis.

Our findings underline the antitumoral potency of PlgK1-5 on hepatoma cells, making it an interesting molecule in the future treatment of hepatocellular carcinomas.

## References

[1] Zhang T, Ding X, Wei D (2010). Sorafenib improves the survival of patients with advanced hepatocellular carcinoma: a meta-analysis of randomized trials. *Anti-Cancer Drugs*.

[2] Rivera L, Pandika M, Bergers G (2014). Escape mechanisms from antiangiogenic therapy: an immune cell's perspective. *Advances in Experimental Medicine and Biology*.

[3] Yardley DA (2013). Drug resistance and the role of combination chemotherapy in improving patient outcomes. *International Journal of Breast Cancer*.

[4] Saraswathy M, Gong S (2013). Different strategies to overcome multidrug resistance in cancer. *Biotechnology Advances*.

[5] Cao Y, Chen A, An SSA (1997). Kringle 5 of plasminogen is a novel inhibitor of endothelial cell growth. *Journal of Biological Chemistry*.

[6] Cao Y, Ji RW, Davidson D (1996). Kringle domains of human angiostatin: characterization of the anti-proliferative activity on endothelial cells. *Journal of Biological Chemistry*.

[7] Perri SR, Nalbantoglu J, Annabi B (2005). Plasminogen kringle 5-engineered glioma cells block migration of tumor-associated macrophages and suppress tumor vascularization and progression. *Cancer Research*.

[8] Schmitz V, Raskopf E, Gonzalez-Carmona MA (2007). Plasminogen fragment K1-5 improves survival in a murine hepatocellular carcinoma model. *Gut*.

[9] Davidson DJ, Haskell C, Majest S (2005). Kringle 5 of human plasminogen induces apoptosis of endothelial and tumor cells through surface-expressed glucose-regulated protein 78. *Cancer Research*.

[10] Schmitz V, Sauerbruch T, Raskopf E (2012). Anti-tumoural effects of PlgK1-5 are directly linked to reduced ICAM expression, resulting in hepatoma cell apoptosis. *International Journal of Colorectal Disease*.

[11] Albini A, Brigati C, Ventura A (2009). Angiostatin anti-angiogenesis requires IL-12: the innate immune system as a key target. *Journal of Translational Medicine*.

[12] Perri SR, Martineau D, François M (2007). Plasminogen Kringle 5 blocks tumor progression by antiangiogenic and proinflammatory pathways. *Molecular Cancer Therapeutics*.

[13] Mauceri HJ, Seetharam S, Beckett MA (2002). Tumor production of angiostatin is enhanced after exposure to TNF-*α*. *International Journal of Cancer*.

[14] Xiao Z, Liu Q, Mao F, Wu J, Lei T (2011). TNF-*α*-induced VEGF and MMP-9 expression promotes hemorrhagic transformation in pituitary adenomas. *International Journal of Molecular Sciences*.

[15] Lu P, Li L, Liu G (2012). Critical role of TNF-alpha-induced macrophage VEGF and iNOS production in the experimental corneal neovascularization. *Investigative Ophthalmology & Visual Science*.

[16] Chen Y-H, Wu H-L, Chen C-K, Huang Y-H, Yang B-C, Wu L-W (2003). Angiostatin antagonizes the action of VEGF-A in human endothelial cells via two distinct pathways. *Biochemical and Biophysical Research Communications*.

[17] Hajitou A, Grignet C, Devy L (2002). The antitumoral effect of endostatin and angiostatin is associated with a down-regulation of vascular endothelial growth factor expression in tumor cells. *The FASEB Journal*.

[18] Schmitz V, Raskopf E, Gonzalez-Carmona M-A (2008). Plasminogen derivatives encoding kringles 1-4 and kringles 1-5 exert indirect antiangiogenic and direct antitumoral effects in experimental lung cancer. *Cancer Investigation*.

[19] Galaup A, Magnon C, Rouffiac V (2005). Full kringles of plasminogen (aa 1-566) mediate complete regression of human MDA-MB-231 breast tumor xenograted in nude mice. *Gene Therapy*.

[20] Bessis N, GarciaCozar FJ, Boissier M-C (2004). Immune responses to gene therapy vectors: influence on vector function and effector mechanisms. *Gene Therapy*.

[21] Cao R, Wu H-L, Veitonmäki N (1999). Suppression of angiogenesis and tumor growth by the inhibitor K1-5 generated by plasmin-mediated proteolysis. *Proceedings of the National Academy of Sciences of the United States of America*.

[22] Llovet JM, Burroughs A, Bruix J (2003). Hepatocellular carcinoma. *The Lancet*.

[23] Genco C, Cabibbo G, Maida M (2013). Treatment of hepatocellular carcinoma: present and future. *Expert Review of Anticancer Therapy*.

[24] Llovet JM, Bruix J (2008). Molecular targeted therapies in hepatocellular carcinoma. *Hepatology*.

[25] Chen Y-H, Wu H-L, Li C (2006). Anti-angiogenesis mediated by angiostatin K1-3, K1-4 and K1-4.5. Involvement of p53, FasL, AKT and mRNA deregulation. *Thrombosis and Haemostasis*.

[26] Weidong-Richard JI, Castellino FJ, Chang Y (1998). Characterization of kringle domains of angiostatin as antagonists of endothelial cell migration, an important process in angiogenesis. *The FASEB Journal*.

[27] Hanford HA, Wong CA, Kassan H (2003). Angiostatin4.5-mediated apoptosis of vascular endothelial cells. *Cancer Research*.

[28] Lee T-Y, Muschal S, Pravda EA, Folkman J, Abdollahi A, Javaherian K (2009). Angiostatin regulates the expression of antiangiogenic and proapoptotic pathways via targeted inhibition of mitochondrial proteins. *Blood*.

[29] (2002). Assessment of adenoviral vector safety and toxicity: report of the National Institutes of Health Recombinant DNA Advisory Committee. *Human Gene Therapy*.

[30] Indraccolo S, Minuzzo S, Gola E (1999). Generation of expression plasmids for angiostatin, endostatin and TIMP-2 for cancer gene therapy. *International Journal of Biological Markers*.

[31] Sun X, Qiao H, Jiang H (2005). Intramuscular delivery of antiangiogenic genes suppresses secondary metastases after removal of primary tumors. *Cancer Gene Therapy*.

[32] Chen Q-R, Kumar D, Stass SA, Mixson AJ (1999). Liposomes complexed to plasmids encoding angiostatin and endostatin inhibit breast cancer in nude mice. *Cancer Research*.

[33] Li J, Yin Q, Wu H (2013). Structural basis of signal transduction in the TNF receptor superfamily. *Advances in Immunology*.

[34] Giraud S, Bienvenu F, Avril S, Gascan H, Heery DM, Coqueret O (2002). Functional interaction of STAT3 transcription factor with the coactivator NcoA/SRC1a. *Journal of Biological Chemistry*.

[35] Aznar S, Valerón PF, Del Rincon SV, Pérez LF, Perona R, Lacal JC (2001). Simultaneous tyrosine and serine phosphorylation of STAT3 transcription factor is involved in RhoA GTPase oncogenic transformation. *Molecular Biology of the Cell*.

[36] Shaik-Dasthagirisaheb YB, Varvara G, Murmura G (2013). Vascular endothelial growth factor (VEGF), mast cells and inflammation. *International Journal of Immunopathology and Pharmacology*.

[37] Azimi-Nezhad M, Stathopoulou MG, Bonnefond A (2013). Associations of vascular endothelial growth factor (VEGF) with adhesion and inflammation molecules in a healthy population. *Cytokine*.

[38] Chung AWY, Hsiang YN, Matzke LA, McManus BM, Van Breemen C, Okon EB (2006). Reduced expression of vascular endothelial growth factor paralleled with the increased angiostatin expression resulting from the upregulated activities of matrix metalloproteinase-2 and -9 in human type 2 diabetic arterial vasculature. *Circulation Research*.

[39] Yang H, Xu Z, Iuvone PM, Grossniklaus HE (2006). Angiostatin decreases cell migration and vascular endothelium growth factor (VEGF) to pigment epithelium derived factor (PEDF) RNA ratio in vitro and in a murine ocular melanoma model. *Molecular Vision*.

